# Mapping associations between anxiety and sleep problems among outpatients in high-altitude areas: a network analysis

**DOI:** 10.1186/s12888-023-04767-z

**Published:** 2023-05-15

**Authors:** Yu Jin, Jiaqi Li, Jing Ye, Xianyu Luo, Amanda Wilson, Lanxue Mu, Pinyi Zhou, Yunhui Lv, Yuanyuan Wang

**Affiliations:** 1 College of Education for the Future, Beijing Normal University, Beijing, China; 2 Key Laboratory of Brain, Cognition and Education Sciences, Ministry of Education, China, School of Psychology, Center for Studies of Psychological Application, Guangdong Key Laboratory of Mental Health and Cognitive Science, South China Normal University, Guangzhou, China; 3 Department of Sleep Medicine, The First People’s Hospital of Yunnan Province, The Affiliated Hospital of Kunming University of Science and Technology, Kunming, China; 4 Division of Psychology, Faculty of Health and Life Sciences, De Montfort University, Leicester, UK

**Keywords:** Anxiety, Sleep problems, High-altitude, Network analysis, Outpatients

## Abstract

**Background:**

Anxiety and sleep problems are common comorbidities among outpatients living in high-altitude areas. Network analysis is a novel method to investigate the interaction and the association between symptoms across diverse disorders. This study used network analysis to investigate the network structure symptoms of anxiety and sleep problems among outpatients in high-altitude areas, and to explore the differences in symptom associations in various sex, age, educational levels and employment groups.

**Methods:**

The data was collected from the Sleep Medicine Center of The First People’s Hospital of Yunnan Province from November 2017 to January 2021 with consecutive recruitment (N = 11,194). Anxiety and sleep problems were measured by the Chinese version of the seven-item Generalized Anxiety Disorder Scale (GAD-7) and the Pittsburgh Sleep Quality Index (PSQI) respectively. Central symptoms were identified based on centrality indices and bridge symptoms were identified with bridge indices. The difference of network structures in various sex, age, educational levels and employment groups were also explored.

**Results:**

Among all the cases, 6,534 (58.37%; 95% CI: 57.45-59.29%) reported experiencing anxiety (GAD-7 total scores ≥ 5), and 7,718 (68.94%; 95% CI: 68.08-69.80%) reported experiencing sleep problems (PSQI total scores ≥ 10). Based on the results of network analysis, among participants, “Nervousness”, “Trouble relaxing”, “Uncontrollable worry” were the most critical central symptoms and bridge symptoms within the anxiety and sleep problems network structure. The adjusted network model after controlling for covariates was significantly correlated with the original (r = 0.75, P = 0.46). Additionally, there were significant differences in edge weights in the comparisons between sex, age and educational levels groups (P < 0.001), while the employed and unemployed groups did not show significant differences in edge weights (P > 0.05).

**Conclusions:**

In the anxiety and sleep problems network model, among outpatients living in high-altitude areas, nervousness, uncontrollable worry, and trouble relaxing were the most central symptoms and bridge symptoms. Moreover, there were significant differences between various sex, age and educational levels. These findings can be used to provide clinical suggestions for psychological interventions and measures targeting to reduce symptoms that exacerbate mental health.

**Supplementary Information:**

The online version contains supplementary material available at 10.1186/s12888-023-04767-z.

## Introduction

The high prevalence of sleep problems increasingly suggests sleep problems are an emerging global epidemic. The potential negative effects reported from sleep problems include low energy and fatigue [1], a decline in physical health [2], reduced work and study efficiency [3], and an increase of negative outcomes in terms of the intentions and behaviors of suicide and self-harm [4]. Previous studies reported that the prevalence of sleep problems varies greatly across countries and regions ranging from around 30–58.4% [5, 6]. In China, the pooled prevalence of sleep problems is reported to be 15–35.9% [7, 8].

The common factors that have been correlated to sleep problems are sex [9, 10], age [11], socioeconomic level [12], educational levels [13], occupation [14, 15]. For example, a meta-analysis of observational studies found that the prevalence of insomnia in females was significantly higher than that in males (OR = 1.58, P < 0.001) [10]. In addition, although several studies reported that older adults suffer from more sleep problems [16, 17], research also suggested that young adults are more vulnerable to chronic sleep deficiency and recurrent circadian disruption than older adults [18]. Another study on older adults in 16 European countries reported that a higher level of education was associated with fewer sleep problems [13]. Moreover, multiple studies have indicated that sleep problems are commonly comorbid with psychiatric problems, specifically the comorbidity could simultaneously appear with accompanied anxiety, and comorbid anxiety could in turn aggravate sleep problems. For example, a study on cognitive-behavioral therapy for insomnia suggested that sleep disorders are more severe in the presence of anxiety [19]. Another survey, that used data collected from a national survey in the United States, revealed that among individuals at high risk for anxiety, the rate of sleep problems was also the highest [20]. In addition, other studies have illustrated that sleep problems and anxiety share a pathogenetic mechanism of hyperarousal caused by dysregulation of neurotransmitter systems, including cholinergic and gamma-aminobutyric acid (GABA) mechanisms [21]. The concomitant relationship between anxiety and sleep problems could be further explained by various sleep or insomnia theories. According to the 3P Behavioral Model [22], of the known three factors (the predisposing factors, the precipitating factors, and the perpetuating factors), anxiety is one of the predisposing factors that is most frequently associated with sleep problems [23, 24]. The psychobiological inhibition model states that stress and anxiety precipitate both physiologic and psychological arousal, and the consequences of this are the occurrence of sleep problems [23, 25]. Furthermore, the hyperarousal model of insomnia [26] presumes that insomnia is a psychobiological disorder, involving psychological disorders and biological imbalances. It proposes that there is a bidirectional connection between anxiety and sleeplessness, which could possibly be illustrated by the depleting biological systems mentioned above. Collectively, the theories and models support that sleep problems are frequently accompanied by anxiety symptoms and vice versa. However, relatively less attention has been paid to investigating the symptomatic relationship of both sides.

Furthermore, an absence of good sleep could be due to geographical factors. Compared to lower altitude settings, there is a high frequency of sleep problems in the pediatric population residing at higher altitudes [27]. Literature suggests that the quality of sleep becomes worse as altitude increases [28, 29], and the quality of sleep of lowland inhabitants who move to live in high-altitude regions remains inferior to that of their native highland counterparts [30]. The Yunnan Province is located in the southwest of China, which is situated in a mountainous area with high altitudes ranging from 3,700 to 5,000 m and above. Therefore, compared with other low-altitude regions, individuals living in the Yunnan Province would have poorer sleep quality due to hypoxia and the decreased partial pressure of inhaled oxygen [31, 32]. Furthermore, studies have found that high-altitude exposure triggers sleep disturbances and is closely associated with anxiety [33]. Thus, investigating associations of symptoms between anxiety and sleep problems is crucial for targeted interventions among outpatients living in Yunnan Province.

Network analysis remains a relatively novel method to observe and predict the relationship between symptoms of various psychiatric disorders [34, 35]. Each of these symptoms constitutes a node correlated to others, and these correlations are named edges [36]. The goal of network analysis is to identify a central node that is highly connected to other nodes, which are called “central symptoms”, and as the name implies that the central symptoms in a network occupy the strongest association with other symptoms [37]. Symptoms that increase the risk of progression from one disorder to the other are called “bridge symptoms”, which are also identified within network analysis [38]. In order to explore the interaction across individual symptoms of diverse disorders, it is also useful to apply network analysis to identify the central symptoms and bridge symptoms between anxiety and sleep problems. Several studies have applied such a novel approach to investigate the relationship between anxiety disorders and poor sleep quality. A study showed that within the anxiety items, “being nervous” had a strong connection with “unable to relax”, which aggravates poor sleep [36]. However, there is a lack of studies focused on the association of symptoms between anxiety and diverse sleep problems. Hence, it is crucial and meaningful to explore the potential implications of these symptoms and provide targeted interventions directed at alleviating particular symptoms.

Few studies exploring the correlation of symptoms between anxiety and sleep problems have been published utilizing the network model. Herein the objectives of this study were to investigate the network structure symptoms of anxiety and sleep problems among outpatients in high-altitude areas, and to explore the differences of symptom associations in various sex, age, educational level and employment groups. These findings could provide better clinical interventions and treatment for individuals with anxiety disorders and sleep problems.

## Methods

### Study design and settings

The data was collected from the Sleep Medicine Center of The First People’s Hospital of Yunnan Province between November 2017 to January 2021 with consecutive recruitment. This hospital is a comprehensive 3 A-grade hospital with strong medical services present in the Yunnan Province. The annual number of outpatient visits to the hospital is more than 2 million, with nearly 90,000 patients discharged each year. The hospital has formed medical consortiums with another 215 hospitals in Yunnan and has supported the development of a network of 20 county-level hospitals in the province. For this study, inclusion criteria included: (1) patients who presented with sleep problems, including losing sleep, snoring, abnormal behaviors during sleep and so forth; and (2) patients with fluency in Mandarin, who can understand the content of the assessment scales accurately and make corresponding answers. Exclusion criteria included: (1) Patients with unstable vital signs or severe physical diseases requiring prompt treatment; (2) Patients with severe psychiatric disorders; and (3) patients affected by other conditions that hinder scale assessment completion (e.g., brain injury). Written informed consent was obtained from all participants included in the study. The research was examined and approved by the ethics committee of The First People’s Hospital of Yunnan Province (number: KHLL2017-KY040).

## Measurements

### Anxiety symptoms

The Chinese version of the seven-item Generalized Anxiety Disorder Scale (GAD-7) was used to measure the severity of anxiety symptoms [39]. The GAD-7 consists of seven items: (1) “Nervousness”; (2) “Uncontrollable worry”; (3) “Excessive worry”; (4) “Trouble relaxing”; (5) “Restlessness”; (6) “Irritability”; and (7) “Feeling afraid”. These items are designed to measure the patient’s health status during the previous two weeks [40]. Previous studies indicate that the GAD-7 is a well-validated tool to screen emotional disorders in the outpatient department of Cardiology before psychiatry visits [41], and the GAD-7 is a reliable and valid screening tool among Chinese people with epilepsy [42]. This scale has proven to have a good sensitivity and specificity (> 85%) when used in China [43].

### Sleep problems

The Chinese version of the Pittsburgh Sleep Quality Index (PSQI) was utilized to assess the severity of sleep problems [44]. The PSQI consists of 19 self-assessment items, which is composed of the following seven parts: (1) “Subjective sleep quality”; (2) “Sleep latency”; (3) “Sleep duration”; (4) “Sleep efficiency”; (5) “Sleep disturbance”; (6) “Use of sleep medication”; and (7) “Daytime dysfunction”. The Chinese version of PSQI is a sensitive, reliable, and valid outcome assessment tool for use in community-based studies of primary sleep problems [45]. Among Chinese populations, a global score on the PSQI that is higher than seven could be regarded as poor sleep quality, which shows good sensitivity of 98.3% and specificity of 90.2% [46]. Moreover, total scores of more than 10 would be considered clinical sleep problems [47].

Each item of both of the two scales was assessed from the range of “0” (not at all) to “3” (almost every day), with higher scores indicating more severe anxiety symptoms. The GAD-7 and the PSQI are well-validated in Chinese populations [42, 48, 49].

## Statistical analyses

### Network estimation

The mean, standard deviation (SD), skewness and kurtosis of all the PSQI and GAD-7 items were computed. In the network analysis, each symptom was defined as a “node” and the relationships among these symptoms were “edges”. For network visualization, the thickness of an edge indicates the strength of the association between nodes. The color of an edge indicates the direction of association. The R package “qgraph” was used to establish a network of relationships between symptoms based on the Graphical Gaussian Model (GGM) [37, 50]. In order to dwindle the number of false edges as well as ameliorate the interpretability of the network, the network was regularized using the graphic least absolute shrinkage and selection operator (LASSO), and in addition, the network selection was based on the Extended Bayesian Information Criterion (EBIC) [37, 51].

The central symptom in the network was determined by calculating the expected influence (EI) [52]. For each node, EI denotes the sum of the weights of the edges in the network connected to that node, scrutinizing the positive and the negative weighting. The bridge EI (bEI) measures the role of a symptom as a linking device between insomnia and anxiety. For a node, its bEI is the sum of its edge weights to all other nodes. In addition, a node’s predictability was estimated using the R package “mgm”, where the variance of all other nodes explains the predictability of a node

Furthermore, in order to test whether the results remained consistent after controlling for covariates, we tested the Expected Influence values of the unadjusted and adjusted networks by the Pearson correlation test. Then, we also conducted an independent-sample t-test to examine whether a difference exists between the two networks, which we then examined again. To evaluate the accuracy and stability of the network, the change range of edge weights, the correlation stability co-efficiency (CS-C) and differences in network attributes were assessed by the non-parametric bootstrapping method [53, 54].

### Network comparison

To determine whether there existed differences in network characteristics in various sex, age, educational levels, and occupation groups, we grouped sex into “male” and “female”; age into “younger adults” and “older adults”; educational level into “above bachelor” and “below bachelor”; occupation into “employed” and “unemployed”. Then we tested whether there existed a statistical difference in global strengths and edge weights of sex, age, educational level, and occupation groups. All comparative tests were conducted by the Network Comparison Test (NCT), a permutation test that assesses differences between two networks, performed with the R-package “NetworkComparisonTest” version 2.0.1 [55, 56]. At first, we divided each variable into two groups and calculated their two networks. Then, based on Global strength and edge weight, the two core characteristics of a network were conducted using the network structure variance test and the global strength invariance test respectively, to test the statistical differences between each of the two groups. The former was conducted on a subsample that was iterated for 1,000 permutations to compare the absolute values of the maximum edge weights’ difference between networks. The latter first compared the distribution of edge weights within each network to determine the characteristics of the network structure, and next, the strength differences per edge between the two networks were compared after correcting for p-values due to multiple trials using Holm-Bonferroni.

## Results

### Descriptive statistics

As seen in Table 1, the sociodemographic characteristics of the sample are presented. Of the 11,194 outpatient cases in the study, 7,385 (65.97%) were younger adults, and 3,809 (34.03%) were older adults. 3,998 (35.70%) were male, and 7,171 (64.10%) were female. Most had received education from a University/College (N:5,269, 47.07%). Among the total sample, 5,188 (46.35%) reported they were employed, 2,640 (23.58%) were retired or unemployed, and 3,366 (30.07%) were students or did not report their current profession. The average score of GAD-7 was 6.97 (SD 5.99), and the average score of PSQI-7 was 8.21 (SD 6.61).

**Table 1:** Description of sociodemographic of the sample (N = 11,194)

Variables	Total N (%)	GAD-7 (cut-off ≥ 5) N (%)	PSQI − 7 (cut-off ≥ 10) N (%)
Age			
Younger adults (＜50 years)	7385 (65.97%)	4649 (71.15%)	4831 (62.59%)
Older adults (≥ 50 years)	3809 (34.03%)	1885 (28.85%)	2887 (37.41%)
Sex			
Male	3998 (35.70%)	2190 (33.52%)	2480 (32.13%)
Female	7171 (64.10%)	4327 (66.22%)	5220 (67.64%)
Missing	25 (0.20%)	17 (0.26%)	18 (0.23%)
Educational levels			
Primary school	1328 (11.80%)	814 (12.46%)	1048 (13.58%)
Secondary school	2075 (18.54%)	1219 (18.66%)	1498 (19.41%)
High school	1771 (15.82%)	1034 (15.82%)	1235 (16.00%)
University/College	5269 (47.07%)	3032 (46.40%)	3446 (44.65%)
Master/Doctorate	412 (3.68%)	233 (3.57%)	243 (3.15%)
Others	47 (0.42%)	29 (0.44%)	36 (0.46%)
Missing	292 (2.60%)	173 (2.65%)	212 (2.75%)
Professional activity			
Employed	5188 (46.35%)	2939 (44.98%)	3539 (45.85%)
Retired or unemployed	2640 (23.58%)	1504 (23.02%)	1799 (23.31%)
Others (Students/Missing)	3366 (30.07%)	2091 (32.00%)	2380 (30.84%)
GAD-7 (Mean; SD)	6.97 (5.99)	10.83 (4.90)	13.65 (4.37)
PSQI-7 (Mean; SD)	8.21 (6.61)	8.26 (6.10)	14.92 (2.97)

Note: N: numbers; GAD-7: The seven-item Generalized Anxiety Disorder Scale; PSQI: The Pittsburgh Sleep Quality Index; SD: Standard deviation

Among all the cases, 6,534 (58.37%; 95% CI: 57.45-59.29%) reported experiencing anxiety (GAD-7 total scores ≥ 5), and 7,718 (68.94%; 95% CI: 68.08-69.80%) reported experiencing sleep problems (PSQI total scores ≥ 10). More younger adults reported anxiety (N:4,649, 71.75%) and sleep problems (N:4,831, 62.59%); females reported more anxiety (N:4,327, 66.22%) and sleep problems (N:5,220, 67.64%) as well. In addition, those whose education levels were University/College reported more anxiety (N:3,032, 46.40%) and sleep problems (N:3,446, 44.45%). Most employed cases showed anxiety (N = 2,939, 44.98%) and sleep problems (N:3,539, 45.85%) when compared to unemployed.

**Fig. 1:**
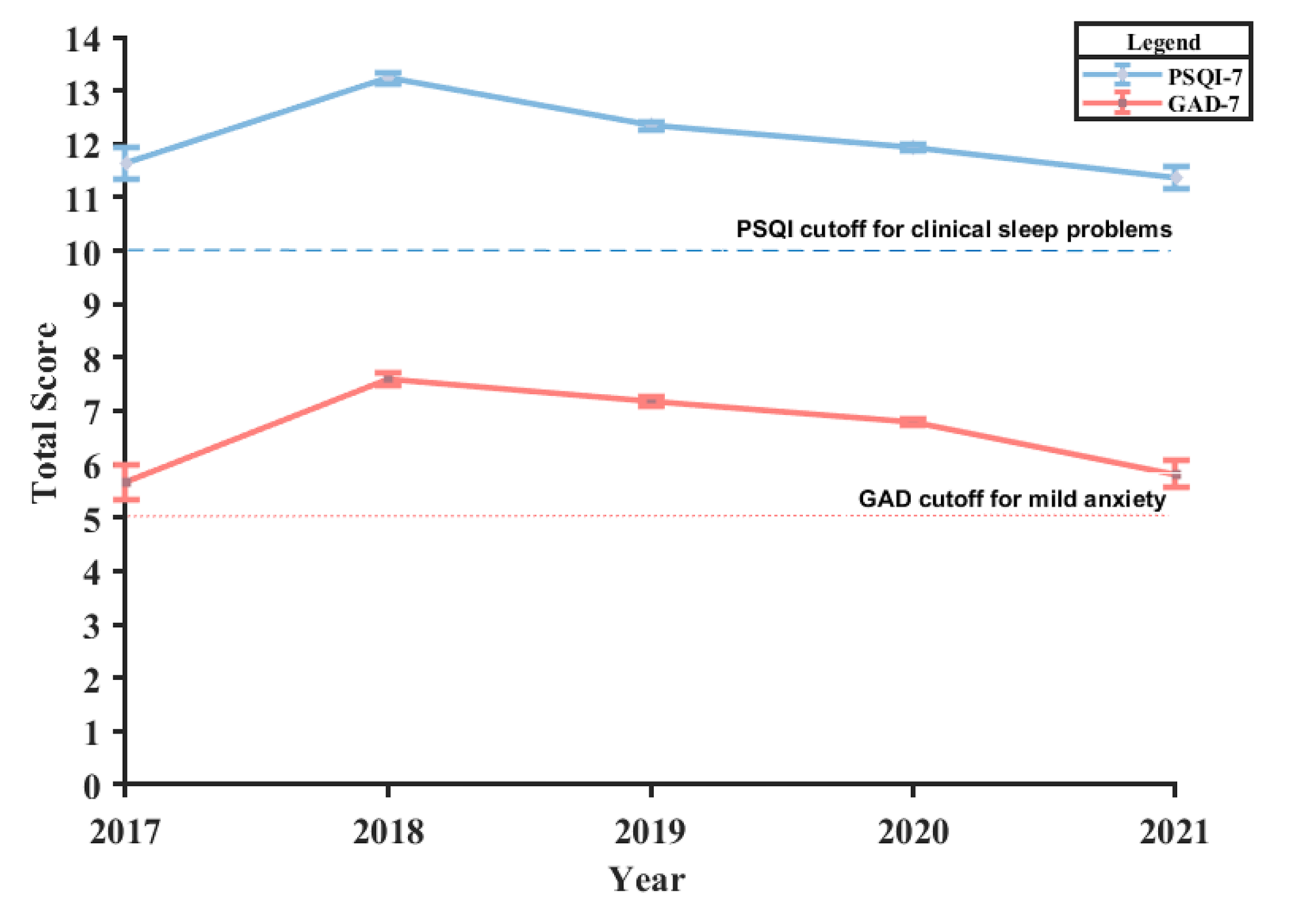
The change of mean values of total scores of PSQI and GAD-7 from 2017 to 2021 Note: GAD-7: The seven-item Generalized Anxiety Disorder Scale; PSQI: the Pittsburgh Sleep Quality Index.

**Fig. 2:**
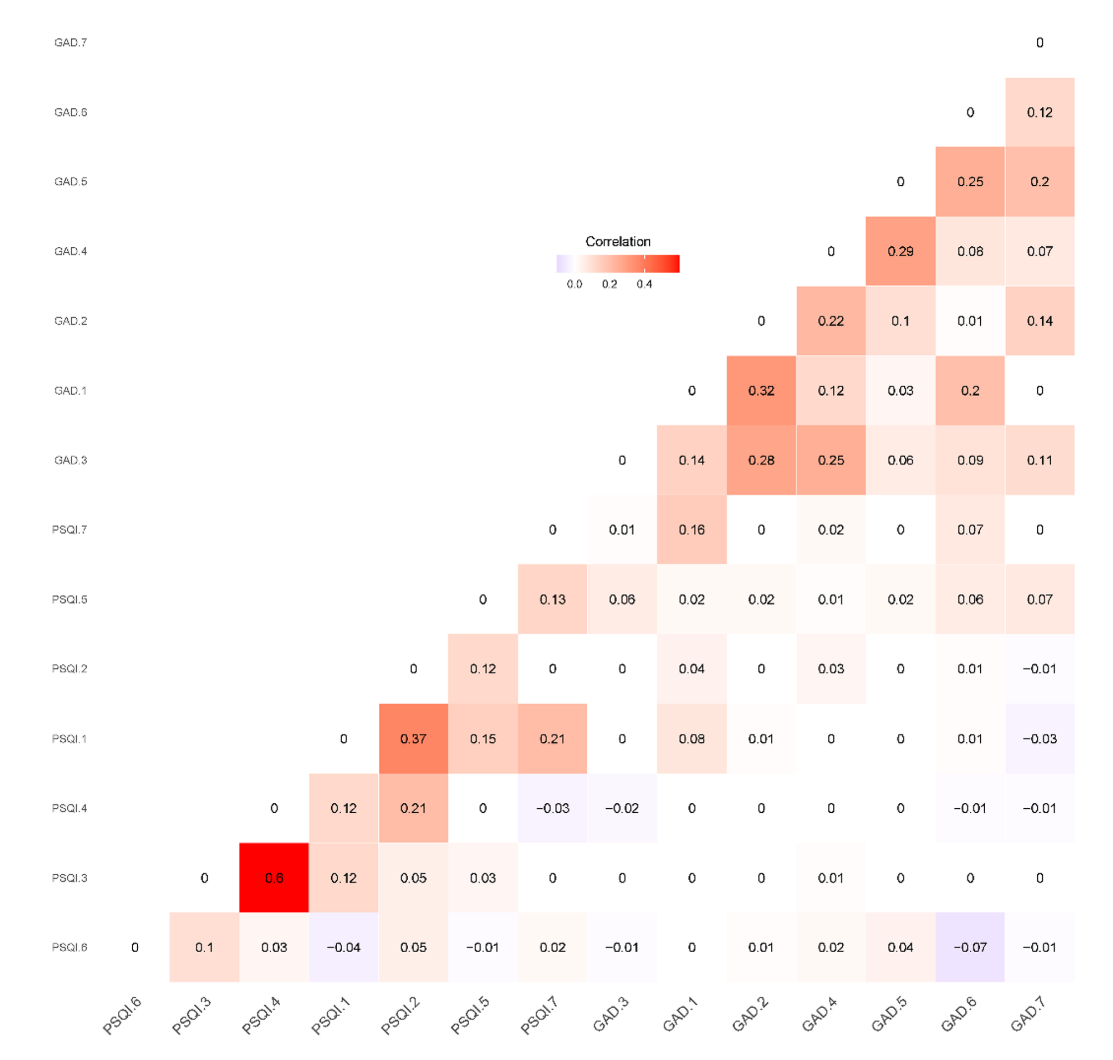
Correlations between GAD-7 items and PSQI components Note: GAD-7: The seven-item Generalized Anxiety Disorder Scale; PSQI: the Pittsburgh Sleep Quality Index

Table 2 displays the basic information of the scales and means, standard deviation (SD), skewness, and kurtosis of each item. Figure 1 shows the change in mean values of total scores for the PSQI and GAD-7 from 2017 to 2021 during the study period. From this figure, it can be found that those outpatients living in high-altitude areas had clinical sleep problems (PSQI total scores ≥ 10) and constantly mild anxiety (GAD-7 total scores ≥ 5). Figure 2 shows the correlations between GAD-7 items and PSQI items.

**Table 2:** Basic information on scales and descriptive item statistics

Scale	Symptoms	Items	Mean	SD	Skewness	Kurtosis
GAD-7	Nervousness	1. Feeling nervous, anxious or on edge	1.35	1.07	0.29	-1.15
	Uncontrollable Worry	2. Not being able to stop or control worrying	1.03	1.05	0.67	-0.78
	Excessive Worry	3. Worrying too much about different things	1.12	1.01	0.57	-0.77
	Trouble relaxing	4. Trouble relaxing	1.03	1.05	0.68	-0.76
	Restlessness	5. Being so restless that it is hard to sit still	0.77	0.98	1.06	-0.03
	Irritability	6. Becoming easily annoyed or irritable	1.07	1	0.63	-0.65
	Feeling afraid	7. Feeling afraid as if something awful might happen	0.59	0.88	1.44	1.13
PSQI	Subjective sleep quality	1. During the past month, how would you rate your sleep quality overall?	1.98	1.02	-0.69	-0.66
	Sleep latency	2. How long (in minutes) has it taken you to fall asleep each night?	1.99	1.07	-0.58	-1.04
		3. During the past month, how often have you had trouble sleeping because a) you cannot get sleep within 30 minutes.				
	Sleep duration	4. How many hours of actual sleep do you get at night?	1.97	1.21	-0.73	-1.1
	Sleep efficiency	5. When have you usually gone to bed?	1.72	1.27	-0.3	-1.6
	Sleep disturbance	6. During the past month, how often have you had trouble sleeping because you:	1.31	0.66	0.42	0.24
		b) wake up in the middle of the night or early morning				
		c) have to get up to use the bathroom				
		d) cannot breathe comfortably				
		e) cough or snore loudly				
		f) feel too could				
		g) feel too hot				
		h) have bad dreams				
		i) have pain				
		j) other reasons				
	Use of sleep medication	7. During the past month, how often have you taken medicine?	1.09	1.35	0.55	-1.56
	Daytime dysfunction	8. During the past month, how often have you had trouble staying awake while driving, eating meals or engaging in social activity?	2.12	0.99	-0.76	-0.63
		9. During the past month, how much of problems has it been for you to keep up enthusiasm to get things done?				

Note: SD: standard deviation; GAD-7: The seven-item Generalized Anxiety Disorder Scale; PSQI: the Pittsburgh Sleep Quality Index

### Network structure

**Fig. 3:**
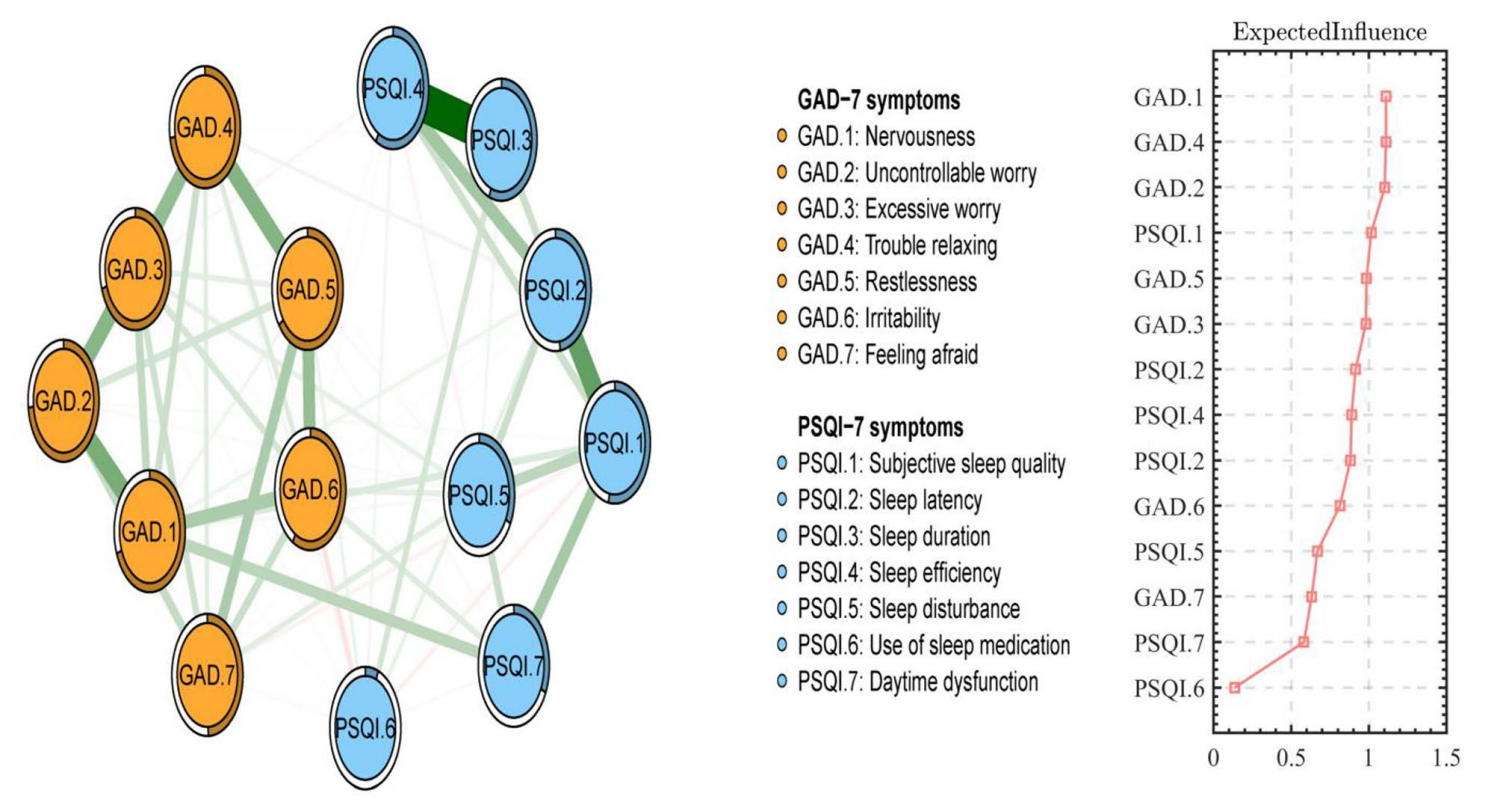
Network structure of anxiety and sleep problems among outpatients (N = 11,194) Note: GAD: The seven-item Generalized Anxiety Disorder Scale; PSQI: the Pittsburgh Sleep Quality Index. Edge weights in the network of anxiety and sleep problems ranged from − 0.07 (GAD.6-PSQI.6) to 0.60 (PSQI.3-PSQI.4)

Figure 3 reveals the network structure of anxiety and sleep problems among outpatients. The predictability of each symptom is illustrated by a circular pie chart. The average predictability of nodes is 0.53, indicating that 53% of the variance of each node on average can be explained by its neighboring nodes. The anxiety and sleep problems network model and structure indexes were re-estimated after controlling for sex, age, educational levels and occupation as covariates. After controlling for covariates the adjusted network model was significantly correlated with the original model (r = 0.75) (Figure S1), and the t-test result also showed that the covariates did not significantly affect the network model (P = 0.46) (Table S1). The edge weights of these two network models are displayed in Table S2 and Table S3.

The symptom relationship network revealed that within the anxiety community, the strongest association was between nodes GAD.1 (“Nervousness”) and GAD.2 (“Uncontrollable worry”), followed by nodes GAD.4 (“Trouble relaxing”), GAD.5 (“Restlessness”), GAD.2 (“Uncontrollable worry”), and GAD.3 (“Excessive worry”). In the sleep problems community, the strongest association was between nodes PSQI.3 (“Sleep duration”) and PSQI.4 (“Sleep efficiency”), followed by the edge between nodes PSQI.1 (“Subjective sleep quality”) and PSQI.2 (“Sleep latency”), and nodes PSQI.2 (“Sleep latency”) and PSQI.4 (“Sleep efficiency”).

In terms of centrality index EI, node GAD.1 (“Nervousness”) had the highest EI centrality in the network, followed by nodes GAD.4 (“Trouble relaxing”), GAD.2 (“Uncontrollable worry”), and PSQI.1 (“Subjective sleep quality”) (As shown in the right part of Fig. 3). This suggests that these four symptoms are the most significant and influential for clinicians to understand the structure of the network model of anxiety and sleep problems.

**Fig. 4:**
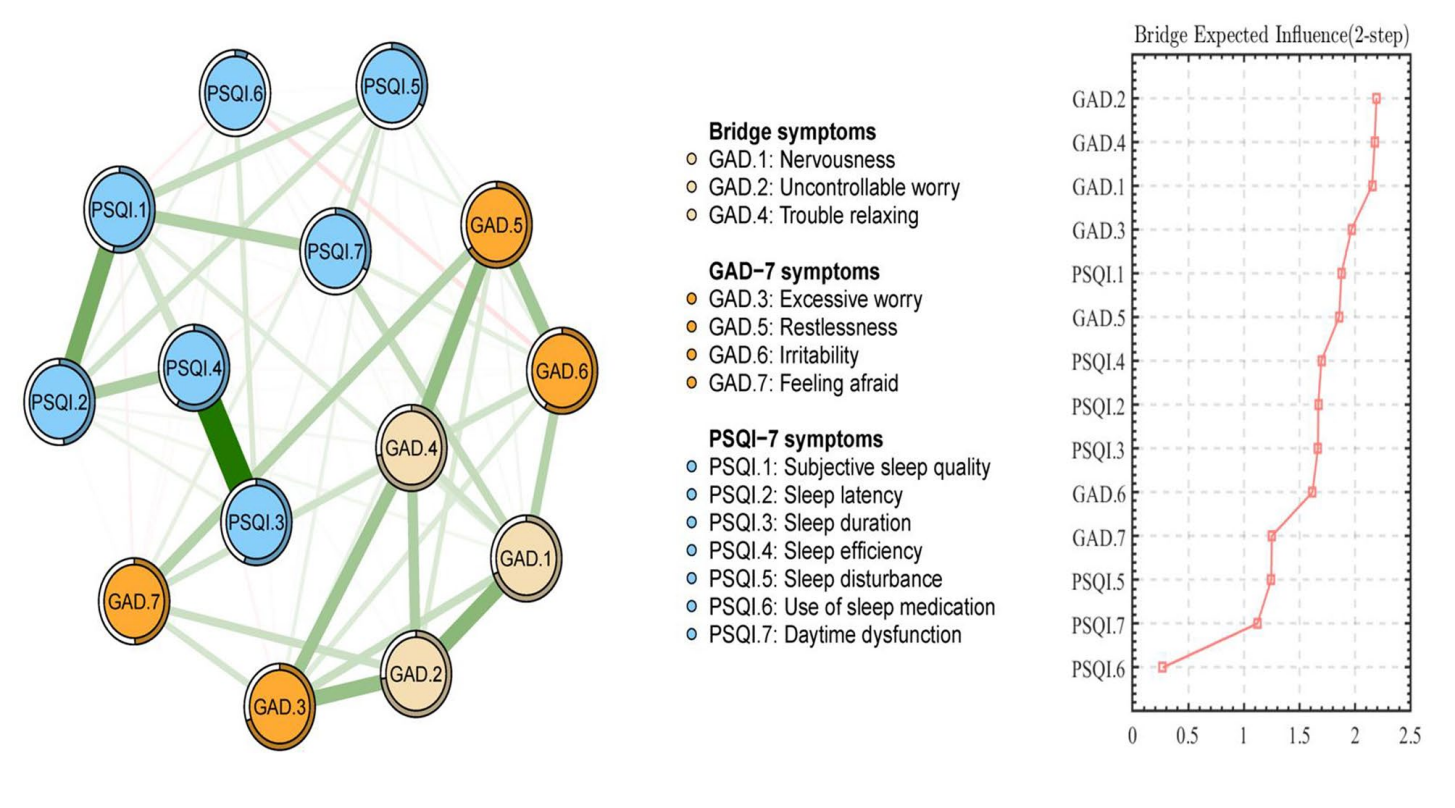
Bridge symptoms between network structure of anxiety and sleep problems among outpatients (N = 11,194) Note: GAD: The seven-item Generalized Anxiety Disorder Scale; Generalized Anxiety Disorders; PSQI: the Pittsburgh Sleep Quality Index. Edge weights in the bridge network of anxiety and sleep problems ranged from − 0.07 (GAD.6-PSQI.6) to 0.60 (PSQI.3-PSQI.4).

For bEI, GAD.2 (“Uncontrollable worry”), GAD.4 (“Trouble relaxing”), and GAD.1 (“Nervousness”) were the most critical bridge symptoms connecting anxiety and sleep problems (As shown in the right part of Fig. 4). In the network structure of anxiety and sleep problems, the connection between GAD.1 (“Nervousness”) and PSQI.7 (“Daytime dysfunction”) (mean edge weight = 0.16) was the strongest edge, followed by the connection between GAD.1 (“Nervousness”) and PSQI.1 (“Subjective sleep quality”) (mean edge weight = 0.08), and the connection between GAD.6 (“Irritability”) and PSQI.7 (“Daytime dysfunction”) (mean edge weight = 0.07).

### Network stability

For the stability of the network, the centrality of EI had a good stability (i.e., CS-C = 0.75), which illustrates that no significant change occurred in the network structure after dropping 75% of the samples (Figure S2). The x-axis represents the percentage of cases in the original sample used at each procedure. The y-axis represents the average of the correlation between the centrality index of the original network and that of the network re-estimated after excluding an increasing number of cases. Each line indicates the correlation among betweenness, closeness, EI, bridge betweenness, bridge closeness, and bridge EI. The results of the bootstrap 95% CI for edges and bootstrapped differences tests for edge weight are found in Figure S3. Simultaneously, the results of eliminating edge weight difference by bootstrapped difference test are illustrated in supplementary Figure S4.

### Network comparison tests for sex, age, educational levels and occupation

According to previous studies, sex, age, educational levels and occupation are associated with anxiety and sleep problems. Comparisons of network models were made between males (N = 3,998) and females (N = 7,171), between younger adults (N = 7,385) and older adults (N =  3,809), between those with educational levels above bachelor (N = 3,620) and below bachelor (N = 7,283), and between employed (N = 6,775) and unemployed (N = 1,609) groups.

**Fig. 5:**
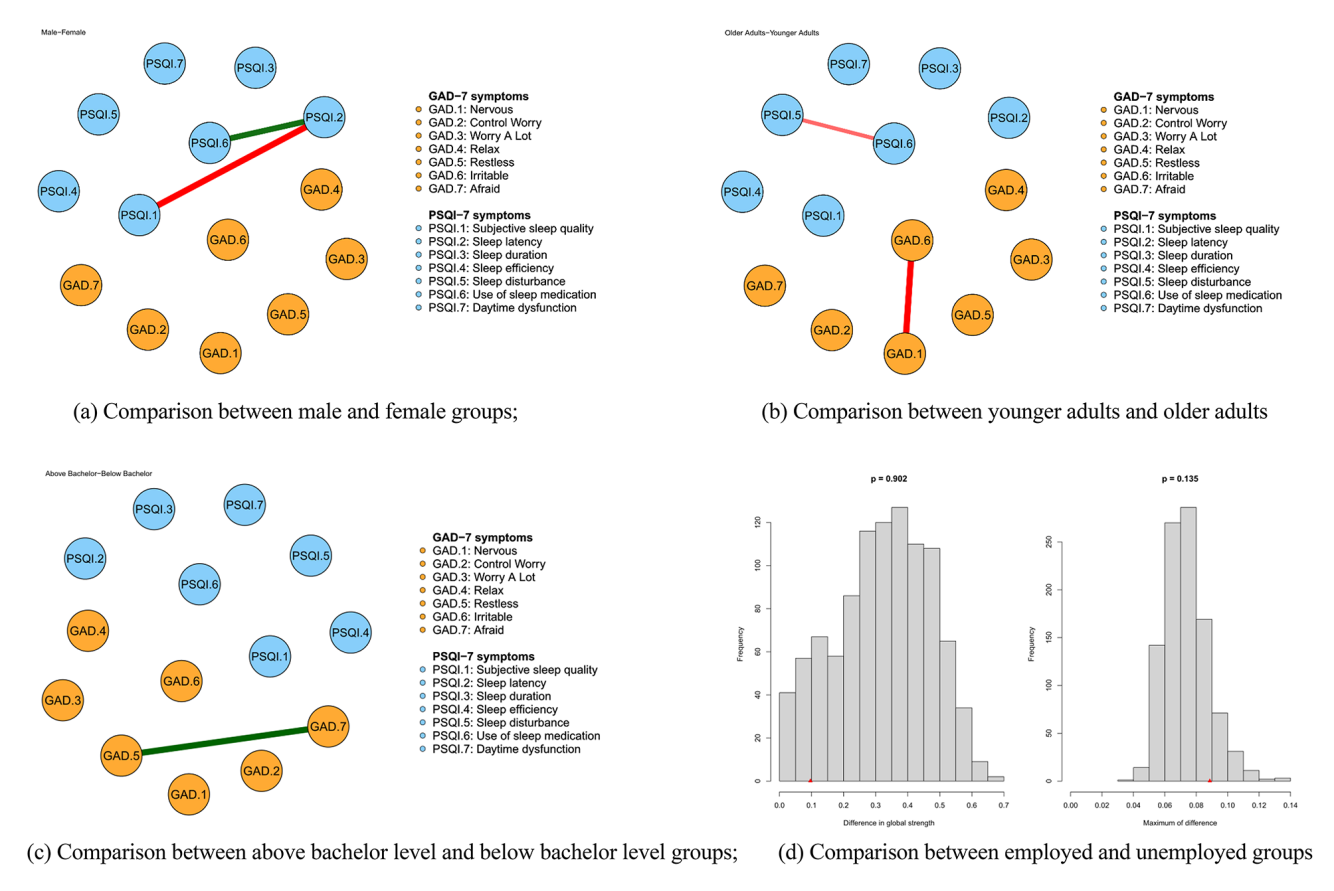
Visual representation of edges with significant difference among the comparisons of sex, age, educational levels and occupation **(a)** Comparison between male and female groups; **(b)** Comparison between younger adults and older adults **(c)** Comparison between above bachelor level and below bachelor level groups; **(d)** Comparison between employed and unemployed groups. Note: GAD: The seven-item Generalized Anxiety Disorder Scale; Generalized Anxiety Disorders; PSQI PSQI: the Pittsburgh Sleep Quality Index

There were significant differences in edge weights in the comparisons between sex, age and educational levels (P < 0.001; Fig. 5 (a), Fig. 5(b), Fig. 5(c), Figure S5-S9; Table S3), while the employed and unemployed groups did not show significant differences in edge weights (Fig. 5 (d)). Moreover, males showed stronger association between PSQI.2 (“Sleep latency”)-PSQI.6 (“Use of sleep medication”), while females showed stronger association between PSQI.2 (“Sleep latency”)-PSQI.1 (“Subjective sleep quality”). In the comparison of age groups, younger adults showed stronger associations than older adults between PSQI.5 (“Sleep disturbance”)-PSQI.6 (“Use of sleep medication”) and GAD.1 (“Nervousness”)-GAD.6 (“Irritability”). In the comparison of educational levels groups, the above bachelor group showed stronger associations between GAD.5 (“Restlessness”)-GAD.7 (“Feeling afraid”) (Table S4).

## Discussion

This is the first study to investigate the symptom correlations of anxiety and sleep problems among outpatients in high-altitude regions using network analysis. In this study, “Nervousness”, “Trouble relaxing”, “Uncontrollable worry”, and “Subjective sleep quality” are the most critical central symptoms within the anxiety and sleep problems network structure. Moreover, besides “Subjective sleep quality”, the other three symptoms were also bridge symptoms connecting anxiety and sleep problems. Thus, these symptoms could be considered the most crucial symptoms and connecting symptoms between anxiety and sleep problems. Additionally, there were significant differences in edge weights in the comparisons between sex, age and educational levels groups, while the employed and unemployed groups did not show significant differences in edge weights.

Sleep problems are widespread among those inhabiting high altitudes [27, 57], which triggers mood disorders, such as anxiety [33]. Based on outpatients from Yunnan Province, a high-altitude region ranging from 3,700 to 5,000 m and above, our results are consistent with previous studies, to a certain degree. Compared to living in lower altitude areas, high-altitude places may induce breathing instability because the oxygen content of blood is reduced, probably leading to sleep disturbances due to a lack of air when sleeping [57]. In addition, during sleep at a high altitude, the hypoxic drive or the response to carbon dioxide (CO_2_) may increase the instability of the control system, thus resulting in periodic breathing, which is considered the main causative factor of sleep problems in people who live at high altitudes above 4,000 m [58]. Furthermore, a large number of psychiatric disorders, such as anxious mood, are associated with long-term accumulated damage to the nervous system due to lack of sleep and high arousal [59].

The strengths of this study are the clinical sample and utilizing a novel network approach to uncover the interacting correlations between anxiety and sleep problems, with visualization. Considering the special geography of the Yunnan Province (with an average elevation is 1,980 m), our results could support clinicians in better understanding symptoms’ associations between anxiety and sleep problems among outpatients living in high-altitude areas. Our results indicated that three anxiety symptoms (nervousness, trouble relaxing, and uncontrollable worry) were the most important symptoms for treatment as well as important for interventions that are aimed at comorbid anxiety and sleep problems. However, these results were different from previous studies conducted in relatively low-altitude regions [60, 61], where our results found that a new symptom “Nervousness” was the most central symptom and connecting symptom. In contrast, several network analyses conducted in flat lands found that trouble relaxing and uncontrollable worry were the core symptoms, and that restlessness and irritability were bridge symptoms between anxiety and sleep problems [60, 61]. Nervousness was the most important symptom of anxiety and sleep problems, among outpatients in high-altitude regions, which is consistent with other research in high-altitude areas. The high-altitude hypoxic environment affects metabolism and the functioning of the nervous system [62]. Living in high-altitude hypoxic areas impairs cognitive function and leads to hypoxic misunderstandings, where normal stimuli at low altitudes, such as slight shortness of breath, are regarded as dangerous stimuli, resulting in nervousness [63]. On the other hand, the cause of anxiety may be due to serotonin and brain bioenergetics depletion in high-altitude areas [64]. Additionally, at high altitudes, brain neurotransmitter systems, such as dopamine, norepinephrine, and glutamate, may potentially enhance susceptibility to nervousness [65]. Cohort studies have found that anxiety scores increased dramatically after exposure to high-altitude regions [33, 66].

Furthermore, the other two symptoms of anxiety (“Trouble relaxing” and “Uncontrollable worry”) were both central and bridge symptoms among outpatients in high-altitude regions, which is consistent with other studies [67] [60] [61]. In other words, those who have the above three anxiety symptoms would trigger sleep problems, which connects anxiety and sleep problems. Thus, these symptoms are important for interventions as well as for the treatment of comorbid anxiety and sleep problems. As previous studies reported, anxiety and sleep problems frequently are co-occurrent [68, 69]. Anxious emotion is commonly regarded as an experience of daily life, which could be a latent alarm bell for individuals. However, when it comes to anxiety disorder, this continuous and false bell could turn into comorbid persistent sleep difficulties [70]. Furthermore, several sleep measurement results also support that a comorbid anxiety disorder is associated with poorer sleep quality [71]. A meta-analysis showed that individuals with anxiety-related disorders had significantly higher sleep disturbances (g = 5.55, 95% CI:4.41–6.70) [72]. Some studies have reported that sleep disturbances are one of the common presentations of anxiety, and common therapies such as transcutaneous vagus nerve stimulation (VNS) and trigeminal nerve stimulation could be utilized to ameliorate both anxiety and sleep problems in the psychiatric field [73–75]. From the neuroendocrine perspective, anxiety or sleep problems are possibly both affected by the lack and deficiency of essential amino acids [76, 77]. A study on sleep and mental health explained that sleep problems could favor an allostatic state that impairs brain plasticity, the emotional, immune, and endocrine pathways, which possibly leads to developing psychiatric disorders [78]. Given that sleep is a vital psychophysiological process for physical and mental health, especially for anxious emotion, and the comorbidity of anxiety with sleep problems, which could bring a diverse degree of consequences for individuals, therefore special attention needs to be paid to the associations between anxiety and sleep problems. All aforementioned factors could have possibly intensified the representation of these central symptoms, which could have increased the risk of outpatients being diagnosed as having anxiety or sleep disorders. Hence, according to network analysis theory, central symptoms underscore the significance of treatment efforts targeting anxiety and sleep problems.

Our findings are inconsistent with earlier statements of network analysis to a certain degree, with our study being based on different population samples. For example, a study reported bridging connections between “Trouble relaxing” and “Sleep problems”, and the link between anxiety and sleep problems was not involved in that bridge [79]. The distinction suggests an underlying possibility, in terms of outpatients seeking medical help when they feel nervous or worry a lot uncontrollably, is that they seemingly present simultaneously with higher sleep problems and anxiety. Meanwhile, according to existing reports, the results could vary across diverse study populations. A previous study showed that there was a higher frequency of sleep problems in the pediatric population at higher altitudes than their counterparts at lower altitude environments [27]. These inconsistencies could be due to the different altitudes of these regions (plateau VS. plain), various measurements of sleep problems (PSQI VS. Insomnia Severity Index), and different study time (normal time VS. during COVID-19), and study sample (outpatients VS. general population). In the future, studies are required to explain these differences.

Meanwhile, our results suggested that an individual’s subjective sleep quality could exert a significant impact on anxiety and sleep problems. Given that the results were from subjective reports by outpatients, this finding was consistent with a previous study of sleep disturbance in anxiety and related disorders, which presented that those individuals with anxiety disorders were more likely to report subjective sleep disturbance [80]. It should be noted that there were non-sufficient direct correlations demonstrated between subjective sleep quality and anxiety or stress among non-clinical populations [80, 81]. Such differences between outpatients and non-clinical population requires further study.

In this network analysis, there was a statistically significant difference in edge weights between various sex, age and educational levels groups. Males showed stronger associations between “Sleep latency” and “Use of sleep medication”, while females showed stronger association between “Sleep latency” and “Subjective sleep quality”. These results were consistent with previous studies. For instance, males are more likely to take medication when suffering from sleep problems, while females are more likely to bear these sleep problems alone [10]. Moreover, females would prefer not to take medication because of worries about the side effects of drugs. However, males would ask for help from the hospitals and take medication to relieve sleep problems [82]. When compared, younger adults showed stronger associations than older adults between “Sleep disturbance” and “Use of sleep medication”, as well as “Nervousness” and “Irritability”. As for the younger adults, they are busy working, caring for families, engaging in social activities and so on, all these factors increase the pressure on younger adults and would increase the risk of sleep disturbance, nervousness and irritability. Furthermore, older adults might suffer from other physical diseases and take medication. Thus, they would prefer not to take more medication for sleep problems due to the worries about negative effects and economic pressure [83]. In addition, results showed that the above bachelor level group showed stronger association between “Restlessness” and “Feeling afraid”. Individuals with education levels above a bachelor’s degree would have more mental pressure due to higher self-demand and self-expectations. Previous studies also found that higher education levels were associated with anxiety because of worries about academic performance, which might increase the risk of restless and being afraid [84, 85]. Therefore, targeted interventions should be applied to these various groups to relieve their anxiety and sleep problems.

Besides, the edge weight between “Sleep duration” and “Sleep efficiency” was the strongest for both males and females, the edge weight of “Nervousness” and “Uncontrollable worry” was stronger for males than for females, while for the edge weight of “Subject sleep quality” and “Sleep latency”, the result was stronger for females than males. For general hospital outpatients, this difference may possibly occur because females encounter more anxious events related to divorced or widowhood and being unemployed, while males appear to have more negative attitudes associated with educational levels [86]. Additionally, in the aspect of age, it was suggested that adults encounter more problems between “Subjective sleep quality” and “Sleep latency” than older adults, possibly because older adults worry less about work or school, while young adults live with more pressure and worries leading to consistently insufficient sleep [11].

Limitations of this study remain to be noted. On the one hand, this investigation was conducted using a hospital database, which could not indicate the causal relationship across the mentioned disorders. While the specific socio-demographic statistics are inadequate, forthcoming studies targeting the casual relationship will be crucial and required. On the other hand, the scales and measurements used could be unlikely to completely avoid subjective reporting distortion, with potential recall bias or other errors. Thirdly, the unbalanced sample size would affect the results, a longitudinal study should be conducted to get more stable network results. Finally, central symptoms and bridge symptoms identified in this study may not be applied to other low-altitude areas.

## Conclusions

In conclusion, this network analysis focused on correlations of symptoms between anxiety and sleep problems in high-altitude regions. Nervousness, uncontrollable worry, and trouble relaxing were the most central symptoms and bridge symptoms, which could provide valuable insight for clinical suggestions for psychological interventions and measures targeting to reduce symptoms that have a deleterious effect on mental health. Moreover, there were significant differences between various sex, age and educational level groups, which suggested that target interventions should be applied to these groups to improve their quality of life.

## Electronic supplementary material

Below is the link to the electronic supplementary material.

Supplementary Material 1 Appendix

## Data Availability

The dataset for this specific manuscript is available from the corresponding author under reasonable request.

## Abbreviations

GAD-7: The seven-item Generalized Anxiety Disorder Scale
PSQI: The Pittsburgh Sleep Quality Index
GABA: Gamma-aminobutyric acid
SD: Standard deviation
GGM: Graphical Gaussian Model
LASSO: Least absolute shrinkage and selection operator
EBIC: Extended Bayesian information criterion
EI: Expected influence
bEI: Bridge expected influence
CIs: Confidence intervals
CS: Correlation stability
NCT: Network Comparison Test
ISI: The Insomnia Severity Index.
