# Supplementary material for: Mapping associations between anxiety and sleep problems among outpatients in high-altitude areas: a network analysis
Source: BMC Psychiatry. 2023 May 15;23:341. doi: 10.1186/s12888-023-04767-z (PMC10184966; doi:10.1186/s12888-023-04767-z)
Supplement: Supplementary file 1 — Supplementary Material 1 Appendix [file 12888_2023_4767_MOESM1_ESM.pdf]

Supplementary Appendix

Figure S1. Visual representation of the network after controlling for age, sex, educational levels, and occupation.

Figure S2. The stability of centrality and bridge centrality indices using case-dropping bootstrap.

Figure S3. Bootstrapped range change of edge weights.

Figure S4. Estimation of edge weight difference by bootstrapped difference test.

Figure S5. Network comparison of male (N=3,998) and female (N=7,171) participants

Figure S6. Comparison of network properties between females and males.

Figure S7. Network comparison of adults (N=7,385) and older adults (N=3,809).

Figure S8. Comparison of network properties between younger adults (N=7,385) and older adults (N=3,809) groups.

Figure S9. Comparison of network properties between above bachelor (N=3,620) and below bachelor (N=7,283) groups.

Table S1. Comparison of the expected influence index values of nodes in the network of anxiety and sleep problem (Unadjusted vs. Adjusted).

Table S2. The partial correlation coefficient between nodes of the network structure of anxiety and sleep problems.

Table S3. The partial correlation coefficient between nodes of the adjusted network structure of anxiety and sleep problems.

Table S4. Significant difference tests for global strength and edge weights of network groups among sex, age, educational levels, and occupation.

Table S5. The value of significant difference on partial correlation coefficient of edges weights in network structures of sex, age, educational level.

Figure S1. Visual representation of the network after controlling for age, sex, educational levels, and occupation.

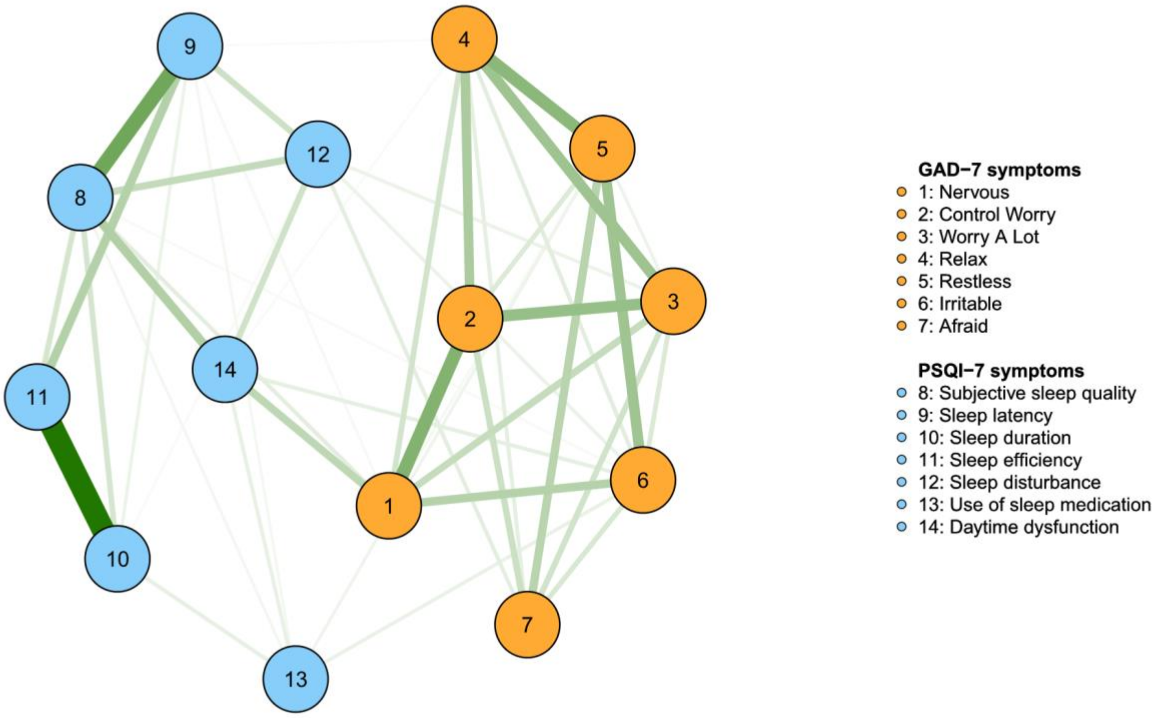

Note: GAD: The seven-item Generalized Anxiety Disorder Scale; PSQI: The Pittsburgh Sleep Quality Index

Figure S2. The stability of centrality and bridge centrality indices using case-dropping bootstrap.

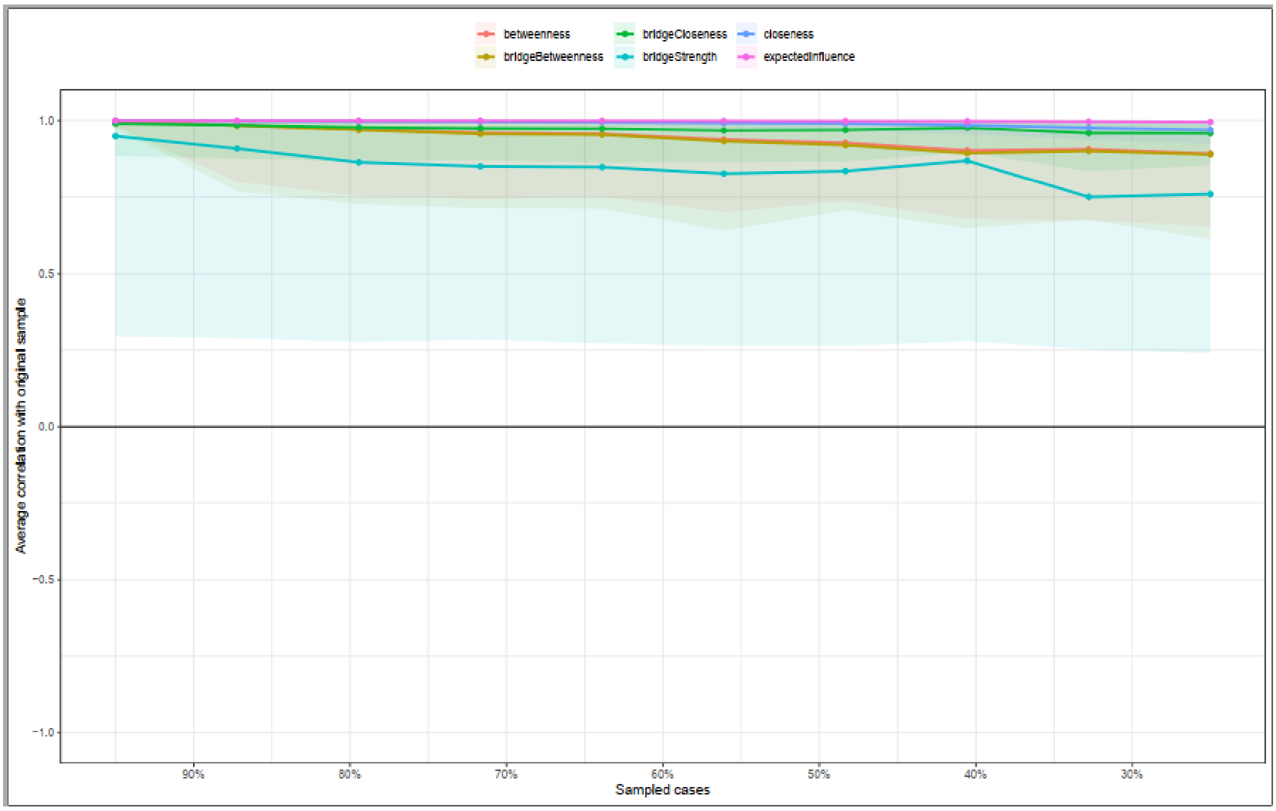

Figure S3. Bootstrapped range change of edge weights.

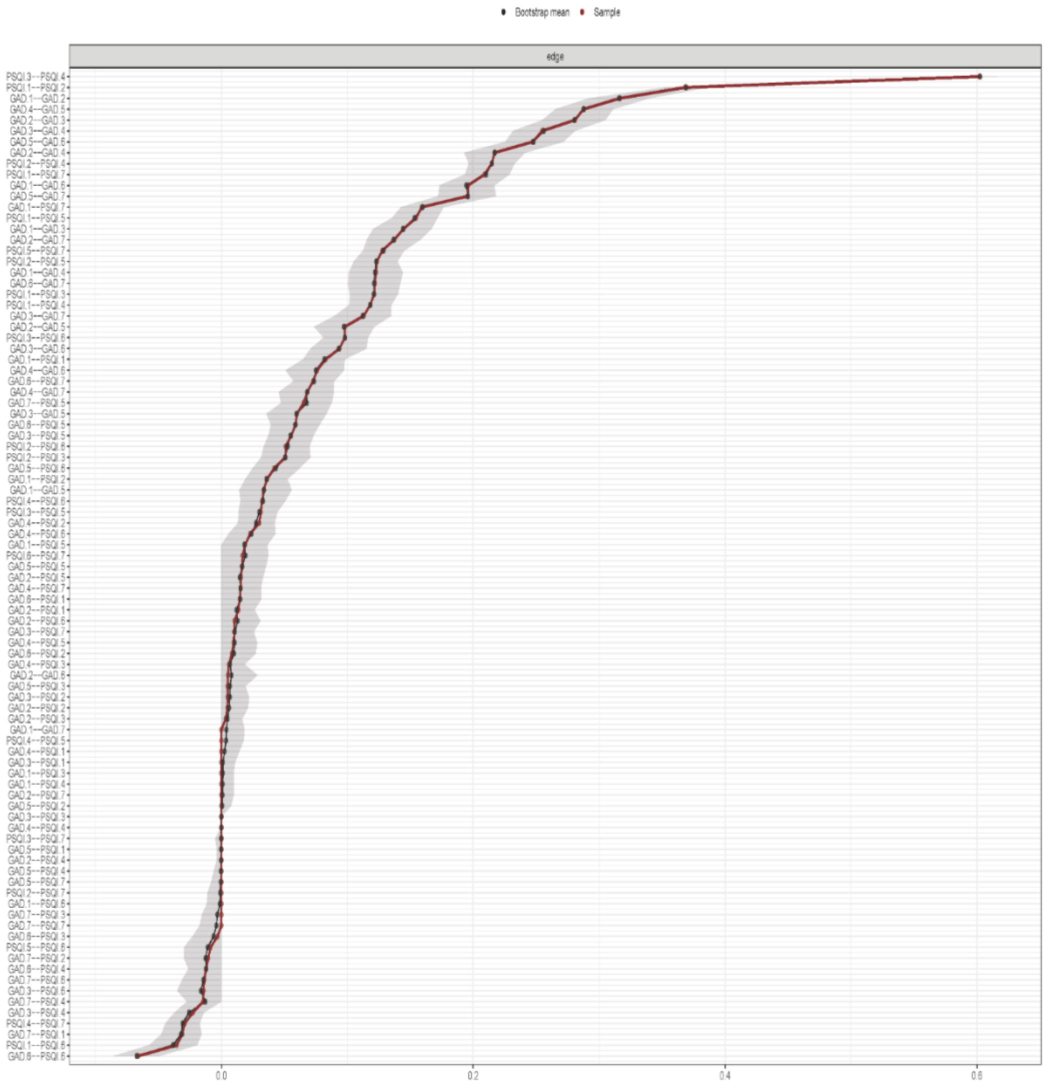

Figure S4. Estimation of edge weight difference by bootstrapped difference test.

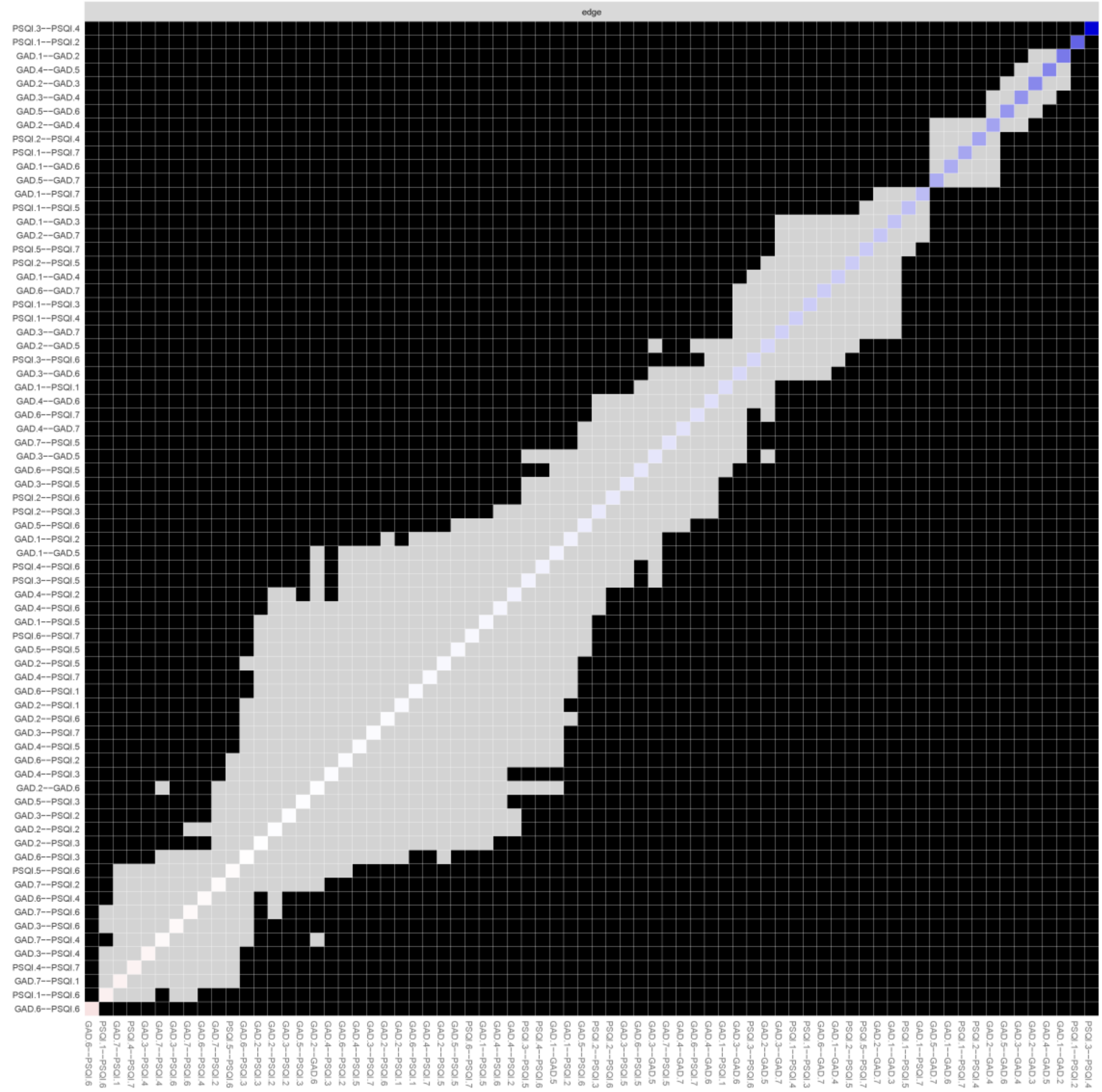

Figure S5. Network comparison of male (N=3,998) and female (N=7,171) participants.

Males (N=3,998).

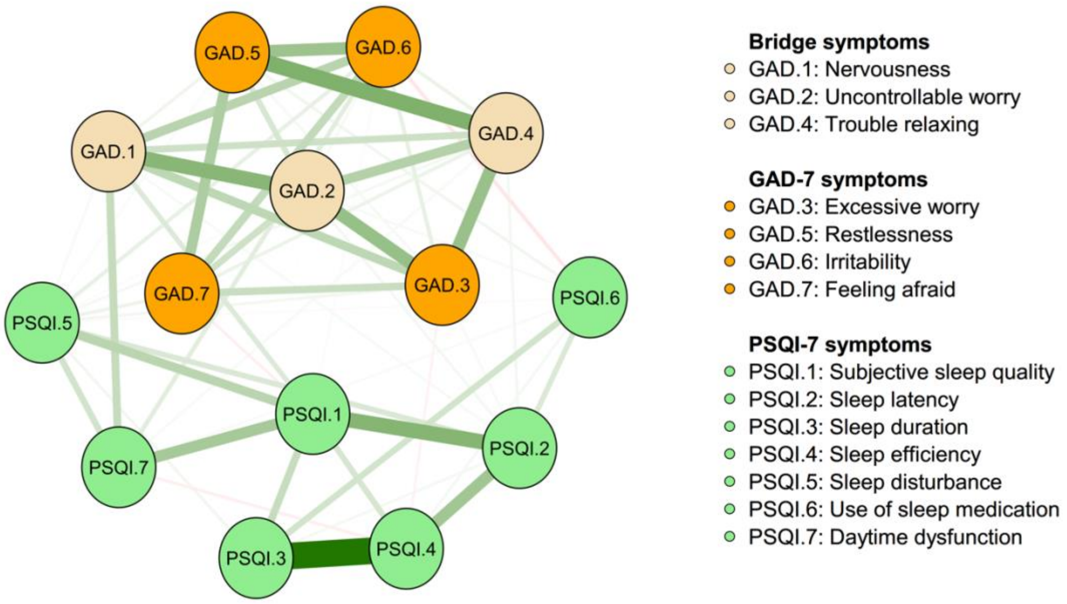

Females (N=7,171)

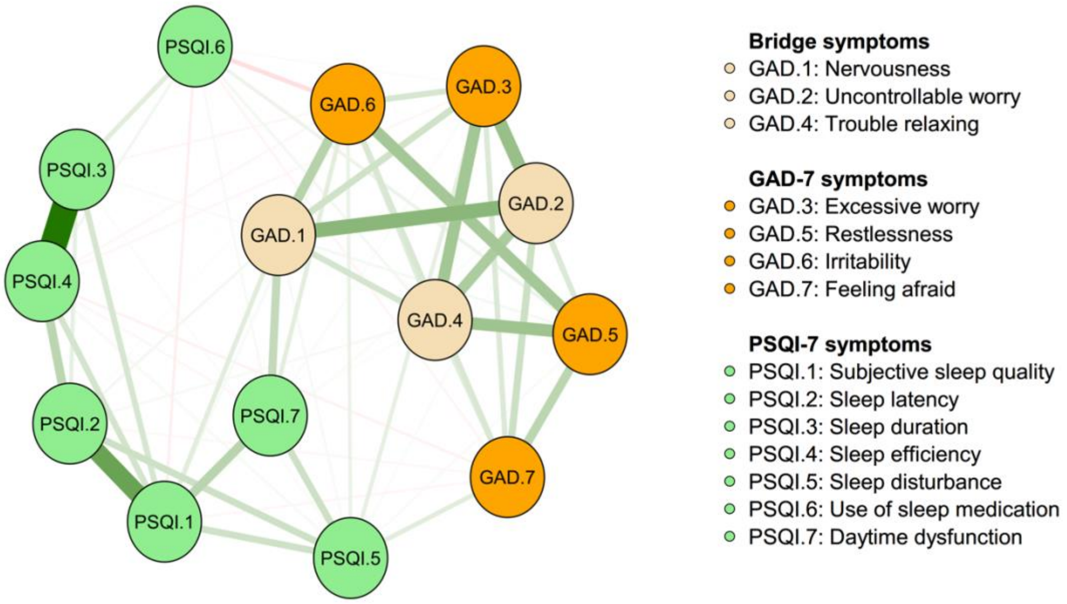

Note: GAD: The seven-item Generalized Anxiety Disorder Scale; PSQI: The Pittsburgh Sleep Quality Index

Figure S6. Comparison of network properties between females and males.

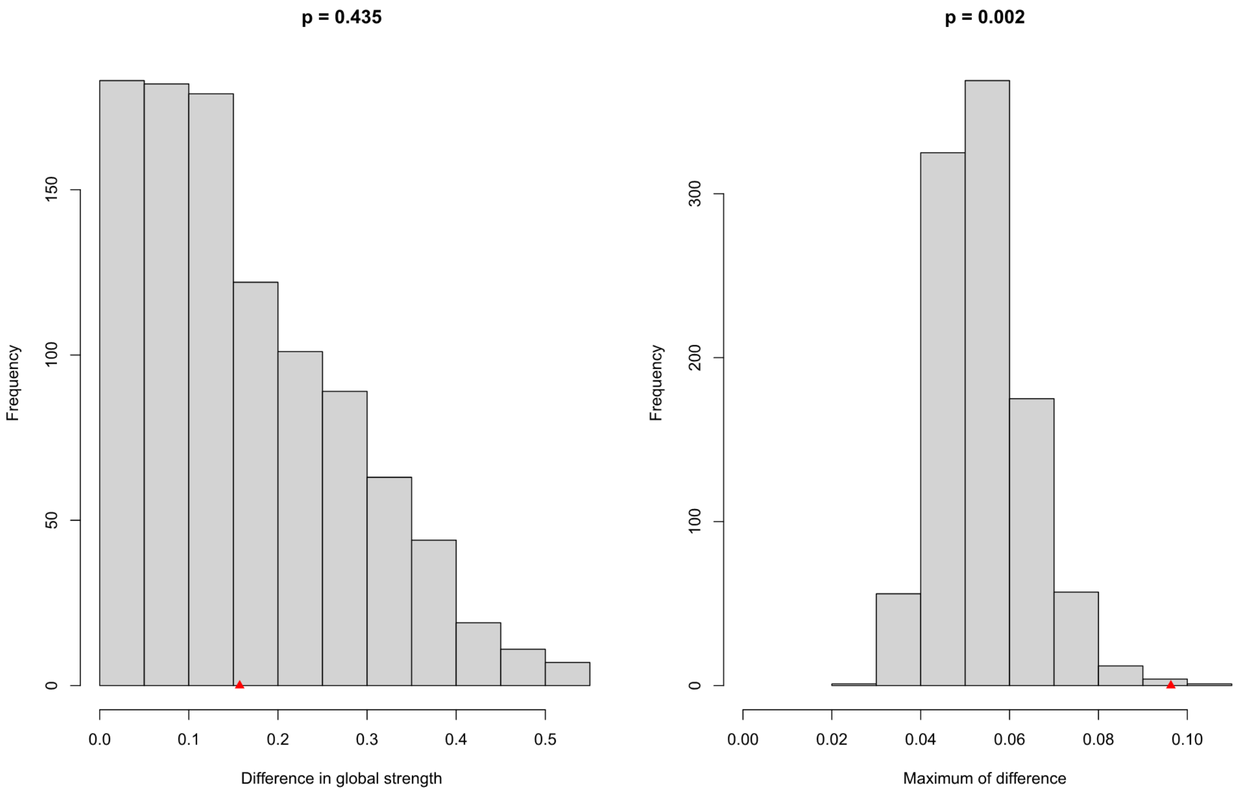

Figure S7. Network comparison of younger adults (N=7,385) and older adults (N=3,809).

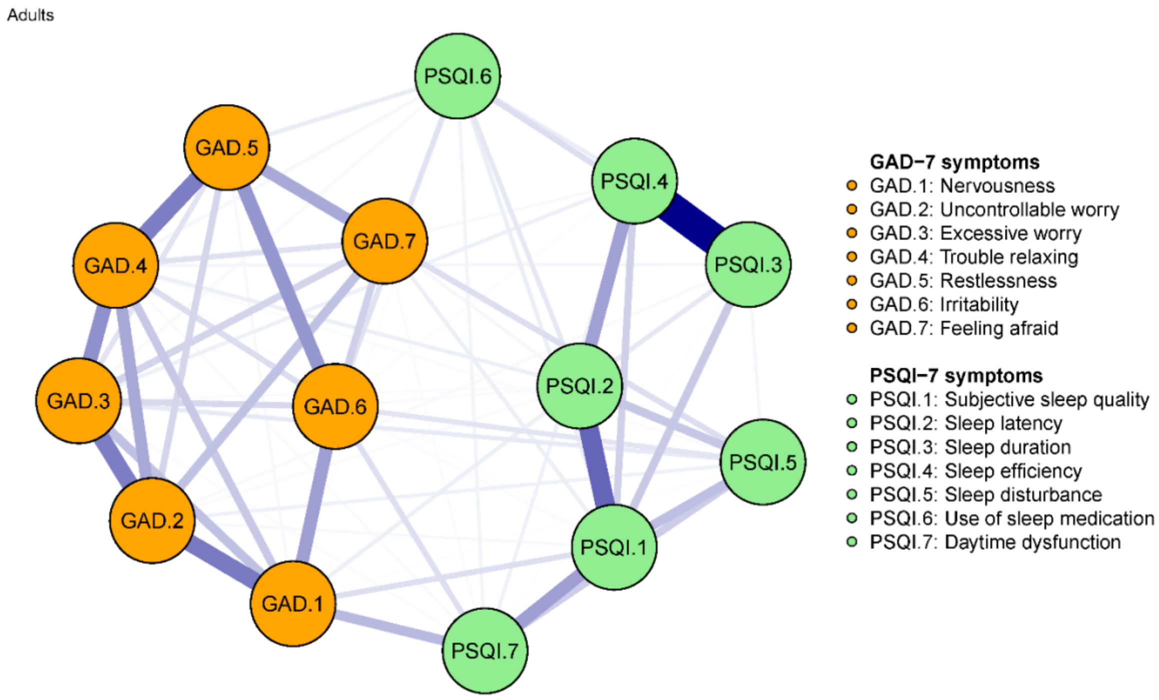

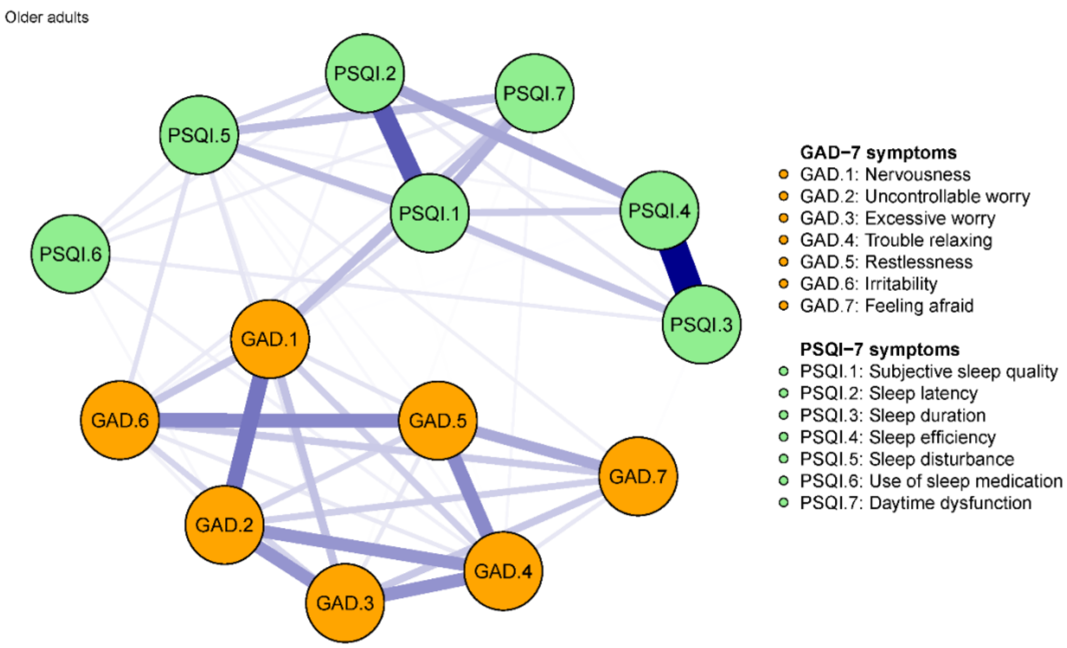

Note: GAD: The seven-item Generalized Anxiety Disorder Scale; PSQI: The Pittsburgh Sleep Quality Index

Figure S8. Comparison of network properties between younger adults (N=7,385) and older adults (N=3,809) groups.

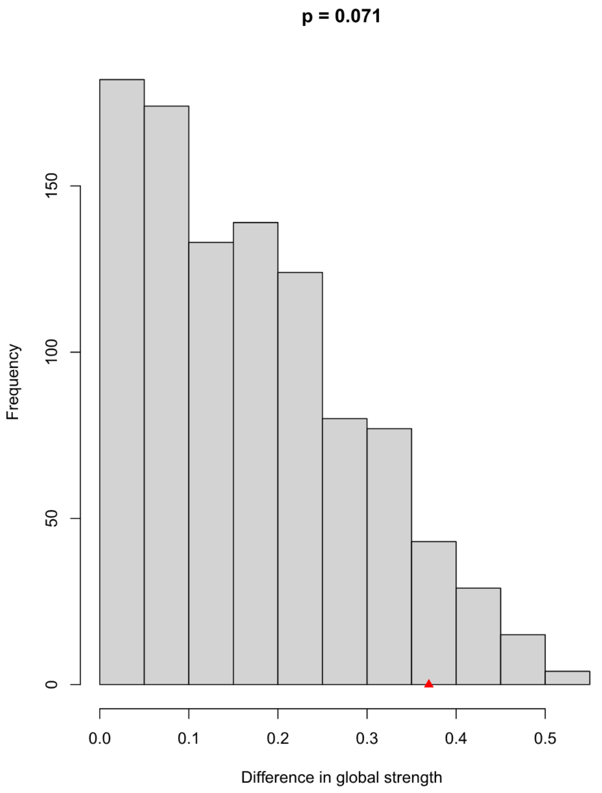

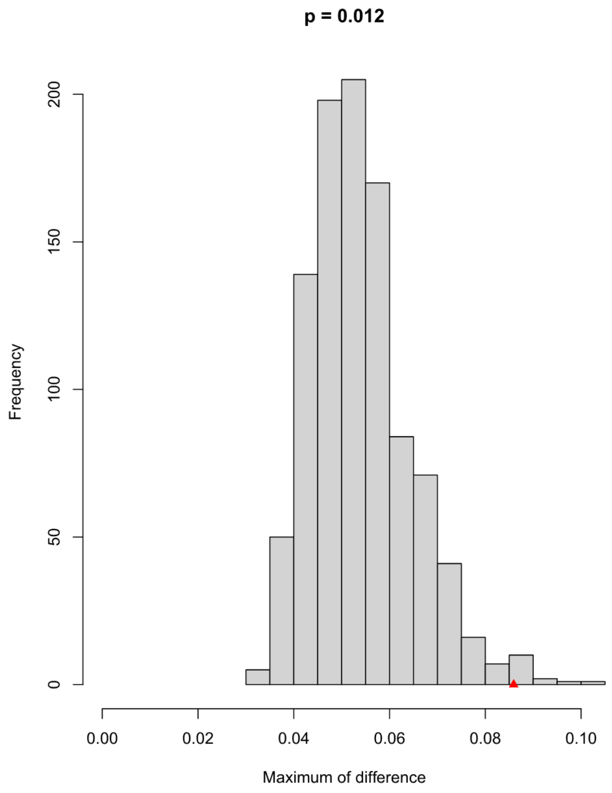

Figure S9. Comparison of network properties between above bachelor's level (N=3,620) and below bachelor's level (N=7,283) groups.

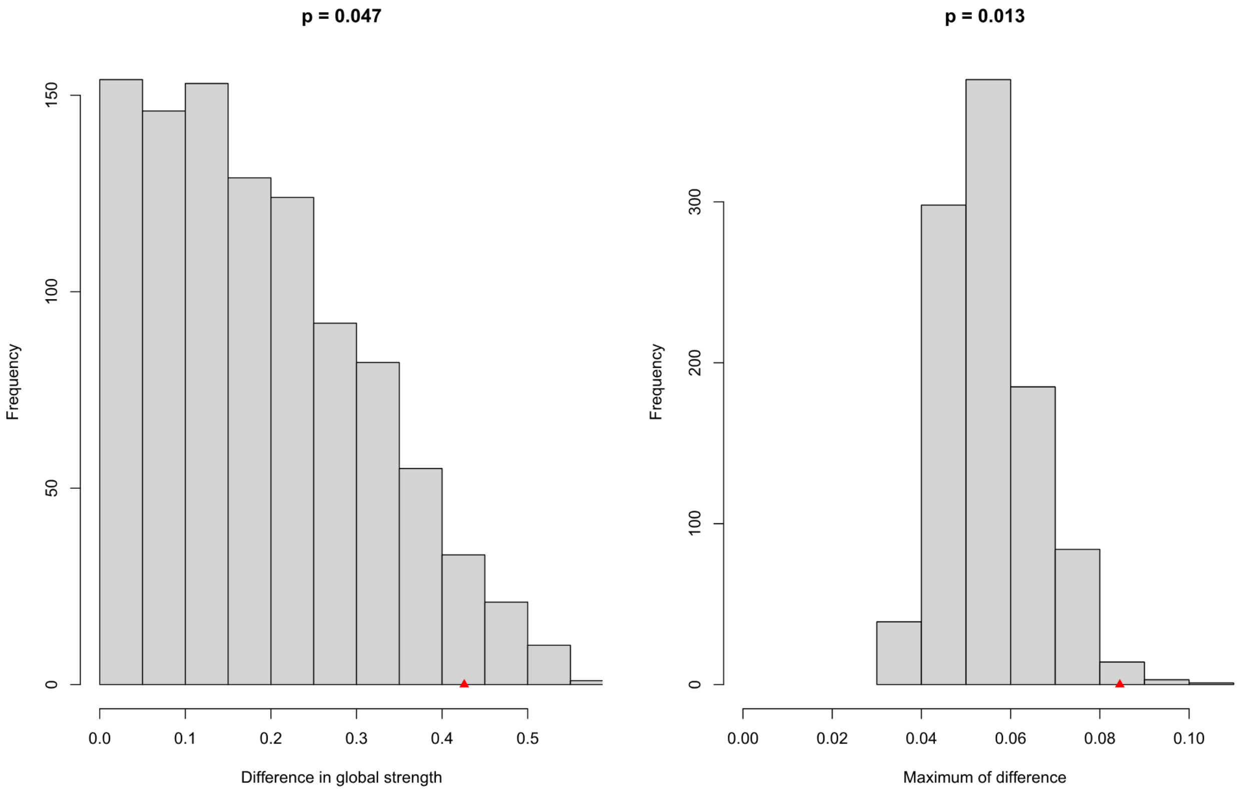

Table S1. Comparison of the expected influence index values of nodes in the network of anxiety and sleep problem (Unadjusted vs. Adjusted).

Node	EI (Unadjusted)	EI (Adjusted)	Pearson correlation	T Value	95% CIs	P Value
GAD.1	1.11	1.10	0.75	0.76	(−0.15, 0.32)	0.46
GAD.2	1.10	0.98				
GAD.3	0.98	1.10				
GAD.4	1.11	0.94				
GAD.5	0.99	0.62				
GAD.6	0.81	0.57				
GAD.7	0.63	0.03				
PSQI.1	1.02	1.11				
PSQI.2	0.88	0.68				
PSQI.3	0.91	1.01				
PSQI.4	0.89	0.90				
PSQI.5	0.67	0.69				
PSQI.6	0.14	0.25				
PSQI.7	0.58	0.65				

Note: GAD: The seven-item Generalized Anxiety Disorder Scale; PSQI: The Pittsburgh Sleep Quality Index; EI, expected influence index; CIs, confidence intervals.

Table S2. The partial correlation coefficient between nodes of the network structure of anxiety and sleep problems.

	GAD.1	GAD.2	GAD.3	GAD.4	GAD.5	GAD.6	GAD.7	PSQI.1	PSQI.2	PSQI.3	PSQI.4	PSQI.5	PSQI.6	PSQI.7
GAD.1	0.00	0.32	0.14	0.12	0.03	0.20	0.00	0.08	0.04	0.00	0.00	0.02	0.00	0.16
GAD.2	0.32	0.00	0.28	0.22	0.10	0.01	0.14	0.01	0.00	0.00	0.00	0.02	0.01	0.00
GAD.3	0.14	0.28	0.00	0.25	0.06	0.09	0.11	0.00	0.00	0.00	-0.02	0.06	-0.01	0.01
GAD.4	0.12	0.22	0.25	0.00	0.29	0.08	0.07	0.00	0.03	0.01	0.00	0.01	0.02	0.02
GAD.5	0.03	0.10	0.06	0.29	0.00	0.25	0.20	0.00	0.00	0.00	0.00	0.02	0.04	0.00
GAD.6	0.20	0.01	0.09	0.08	0.25	0.00	0.12	0.01	0.01	0.00	-0.01	0.06	-0.07	0.07
GAD.7	0.00	0.14	0.11	0.07	0.20	0.12	0.00	-0.03	-0.01	0.00	-0.01	0.07	-0.01	0.00
PSQI.1	0.08	0.01	0.00	0.00	0.00	0.01	-0.03	0.00	0.37	0.12	0.12	0.15	-0.04	0.21
PSQI.2	0.04	0.00	0.00	0.03	0.00	0.01	-0.01	0.37	0.00	0.05	0.21	0.12	0.05	0.00
PSQI.3	0.00	0.00	0.00	0.01	0.00	0.00	0.00	0.12	0.05	0.00	0.60	0.03	0.10	0.00
PSQI.4	0.00	0.00	-0.02	0.00	0.00	-0.01	-0.01	0.12	0.21	0.60	0.00	0.00	0.03	-0.03
PSQI.5	0.02	0.02	0.06	0.01	0.02	0.06	0.07	0.15	0.12	0.03	0.00	0.00	-0.01	0.13
PSQI.6	0.00	0.01	-0.01	0.02	0.04	-0.07	-0.01	-0.04	0.05	0.10	0.03	-0.01	0.00	0.02
PSQI.7	0.16	0.00	0.01	0.02	0.00	0.07	0.00	0.21	0.00	0.00	-0.03	0.13	0.02	0.00

Note: GAD: The seven-item Generalized Anxiety Disorder Scale; PSQI: The Pittsburgh Sleep Quality Index.

Table S3. The partial correlation coefficient between nodes of the adjusted network structure of anxiety and sleep problems.

	GAD.1	GAD.2	GAD.3	GAD.4	GAD.5	GAD.6	GAD.7	PSQI.1	PSQI.2	PSQI.3	PSQI.4	PSQI.5	PSQI.6	PSQI.7
GAD.1	0.00	0.32	0.15	0.11	0.03	0.19	0.00	0.07	0.02	0.00	0.00	0.00	0.00	0.17
GAD.2	0.32	0.00	0.27	0.22	0.09	0.00	0.13	0.00	0.00	0.00	0.00	0.00	0.00	0.00
GAD.3	0.15	0.27	0.00	0.25	0.05	0.09	0.12	0.00	0.00	0.00	0.00	0.05	0.00	0.00
GAD.4	0.11	0.22	0.25	0.00	0.30	0.07	0.07	0.00	0.02	0.00	0.00	0.00	0.00	0.02
GAD.5	0.03	0.09	0.05	0.30	0.00	0.25	0.19	0.00	0.00	0.00	0.00	0.00	0.04	0.00
GAD.6	0.19	0.00	0.09	0.07	0.25	0.00	0.10	0.02	0.00	0.00	0.00	0.05	0.06	0.07
GAD.7	0.00	0.13	0.12	0.07	0.19	0.10	0.00	0.00	0.00	0.00	0.00	0.07	0.00	0.00
PSQI.1	0.07	0.00	0.00	0.00	0.00	0.02	0.00	0.00	0.38	0.11	0.11	0.15	0.03	0.20
PSQI.2	0.02	0.00	0.00	0.02	0.00	0.00	0.00	0.38	0.00	0.05	0.20	0.13	0.03	0.00
PSQI.3	0.00	0.00	0.00	0.00	0.00	0.00	0.00	0.11	0.05	0.00	0.58	0.02	0.06	0.00
PSQI.4	0.00	0.00	0.00	0.00	0.00	0.00	0.00	0.11	0.20	0.58	0.00	0.00	0.00	0.00
PSQI.5	0.00	0.00	0.05	0.00	0.00	0.05	0.07	0.15	0.13	0.02	0.00	0.00	0.00	0.13
PSQI.6	0.00	0.00	0.00	0.00	0.04	0.06	0.00	0.03	0.03	0.06	0.00	0.00	0.00	0.06
PSQI.7	0.17	0.00	0.00	0.02	0.00	0.07	0.00	0.20	0.00	0.00	0.00	0.13	0.06	0.00

Note: GAD: The seven-item Generalized Anxiety Disorder Scale; PSQI: The Pittsburgh Sleep Quality Index.

Table S4. Significant difference tests for global strength and edge weights of network groups among sex, age, educational levels, and occupation.

	Global Strength			Edge Weight	
	M Statistics	Test statistics	P value	Test statistics	P value
Sex					
Male group	6.31	0.16	0.435	0.10	**< 0.01**
Female group	6.46				
Age					
Older adult	6.07	0.37	0.071	0.09	**< 0.05**
Younger adult	6.44				
Educational levels					
Above bachelor	6.09	0.43	0.047	0.08	**0.013**
Below bachelor	6.52				
Occupation					
Employed	6.58	0.10	0.902	0.09	0.135
Unemployed	6.48				

Table S5. The value of significant difference on partial correlation coefficient of edges weights in network structures of sex, age, educational level.

	Sex	Age	Educational Levels
PSQI.1-PSQI.2	-0.10***		
PSQI.2-PSQI.6	0.09***		
GAD.1-GAD.6		-0.09***	
PSQI.5-PSQI.6		-0.05***	
GAD.5-GAD.7			0.08***

Note: ***, P<0.001; GAD, Generalized Anxiety Disorders; PSQI, Pittsburgh Sleep Quality Index; Other associations between symptoms were not reported because of P value large than 0.001.
